# Technical Sprinting in the Early Phase of Hamstring Injury Rehabilitation to Accelerate Return to Full Participation in Track and Field Athletes: A Comparative Study of Two Rehabilitation Strategies

**DOI:** 10.7759/cureus.58268

**Published:** 2024-04-14

**Authors:** Nilesh Makwana, Jayesh Bane, Lipsa Ray, Bhagyashree Karkera, James Hillier

**Affiliations:** 1 Physical Medicine and Rehabilitation Department, Reliance Foundation, Mumbai, IND; 2 Physical Medicine and Rehabilitation Department, Odisha Reliance Foundation Athletics High Performance Centre, Bhubaneshwar, IND; 3 Coaching Department, Reliance Foundation, Mumbai, IND

**Keywords:** sports rehabilitation, track and field, sports performance, sprint, hamstring injury

## Abstract

Introduction: Hamstring injuries are common in track and field athletes with a higher incidence in males than females. It causes a significant loss in training time and a decline in performance. This study evaluated rehabilitation strategies to accelerate return to full participation following hamstring injury.

Methods: Thirty-three athletes (22 males; 11 females) were screened from November 2021 to October 2023 until their final major competition. Out of these, 17 athletes with hamstring injuries were included in this study which were further divided into two groups, A (n=8) and B (n=9), using stratified random sampling with single blinding. Group A received technical sprints using mini hurdles as part of their training from the early stages of rehabilitation, while Group B underwent high-volume low-intensity rehabilitation before progressing to sprints. The progress of each group was monitored on a weekly basis. The average time loss was calculated using Microsoft Excel (Microsoft® Corp., Redmond, WA) and Google Forms (Google, Inc., Mountain View, CA) with built-in validation.

Results: The two groups demonstrated a significant difference in recovery times. In group A, the length of hamstring tenderness (LHT) improved from 9 ± 2.7 (95% CI 2.27) to 0.15 ± 0.3 (95% CI 0.62), active total knee extension (ATKE) from 161.8 ± 7.1 (95% CI 5.95) to 175.4 ± 2 (95% CI 2.3), and Numeric Pain Rating Scale (NPRS) in the isometric test from 5.6 ± 1.09 (95% CI 0.88) to 0.6 ± 0.5 (95% CI 0.63) with p<0.05, and in Group B, LHT improved from 6.8 ± 2.1 (95% CI 1.62) to 0.6 ± 0.7 (95% CI 0.55), ATKE improved from 168.7 ± 8.2 (95% CI 6.3) to 178.7 ± 2.7 (95% CI 2.06) and NPRS with resisted isometric test improved from 6 ± 1.4 (95% CI 1.08) to 0.8 ± 0.7 (95% CI 0.51) with p<0.05. However, Group A took an average of 3.55 weeks (1.22 SD 95% CI 1.08) and Group B took an average of 4.53 weeks (1.98 SD, 95% CI 1.52) to resume full participation. Three athletes from Group A and six athletes from Group B experienced hamstring tightness during the competition, two athletes from Group B were forced to withdraw from the competition due to hamstring reinjury.

Conclusions: The findings indicate that an early technical sprint program can facilitate an early return to full participation. This research can be a guide toward accelerated and integrated hamstring injury rehabilitation among track and field athletes.

## Introduction

Sprinting is a sport that requires exceptional strength and speed. The most prevalent muscle injury in high-intensity sprinting is the hamstring which comprises 17% of all injuries in track and field. Studies have consistently demonstrated that male athletes are at a higher risk of injury than female athletes, particularly in the hamstring region [1]. These injuries often result in substantial loss of training time and have a detrimental impact on athletic performance. The hamstring reinjury rate is high. Hence, a good hamstring injury rehabilitation program is imperative [2,3].

The mechanism of hamstring injury is inconclusive. However, there have been various studies on the biomechanical considerations for sprinting. Some studies suggest that hamstring injuries primarily occur during the early stance phase [4,5], while others indicate the swing phase [6-12]. A forward lean during sprinting has also been identified as a risk factor for hamstring injury [5], while another study found that both the early stance and swing phases presented as equal risk factors for injury [13]. Few other predisposing factors for hamstring injuries in sprinting have also been studied which include lumbar spine or sacroiliac joint pathologies without neural issues [14-16], lack of flexibility in the hamstring and lumbopelvic complex [17-20], poor conditioning, overtraining, undertraining, excessive stretching, and muscle imbalances [19,21-24].

Additionally, sprint kinematics play a significant role in hamstring injuries and performance. Previous research has evaluated the effect of physiotherapy, strength training, and coaching cues on improving posture and biomechanics, which may contribute to a reduced risk of hamstring injuries during high-intensity sprinting [25]. The rehabilitation program for hamstring injuries has been extensively researched, however, there is a lack of data regarding effective strategies for promoting an early return to full participation following hamstring injury in track and field athletes. Therefore, this study aims to investigate the effectiveness of incorporating technical sprinting in the early phase of hamstring injury rehabilitation to accelerate return to full participation as compared to isolated exercises in track and field athletes.

## Materials and methods

Samples

A prospective screening of 33 athletes (22 male and 11 female) was done between November 2021 and October 2023, out of which 17 hamstring injuries were recorded and included in this study. The injured athletes were then divided into two groups based on a coaching strategy using stratified random sampling using single blinding. Group A comprises athletes with a mean age of 21.2 ± 3.2 years, height of 175.9 ± 10.2 cm, and mass of 62.5 ± 11.0 kg, and Group B comprises athletes with a mean age of 20.31 ± 3.19 years, height of 173.21 ± 8.25 cm, and mass of 61.88 ± 7.9, with no statistical baseline difference among groups p>0.05. Athletes who included sprinting as part of their training program were included in this study and competed in national-level competitions organized by the National Federation. The athlete characteristics are presented in Table 1.

**Table 1:** The athletes’ characteristics according to coaching groups The table demonstrates the two different coaching groups A and B with a variety of events. The above athletes performed sprints as part of their conditioning and speed-related training program. It included a total of 33 athletes who were monitored from October 2021 till November 2023 from Odisha Reliance Foundation Athletics High Performance Centre, Bhubaneshwar.

Coaching Groups	Events	Male	Female	Total
Coaching Group A (n=16)	100/200 m	3	1	4
	100/110 m Hurdle	4	3	7
	400 m Hurdle	1	1	2
	400 m	1	0	1
	High Jump	2	0	2
Coaching Group B (n=17)	100/200m	7	1	8
	100/110 m Hurdle	1	2	3
	400 m Hurdle	1	2	3
	Decathlon/Heptathlon	1	1	2
	Triple Jump	1	0	1

The athletes' training blocks

The season was structured into several training periods, known as training blocks, which typically consisted of three weeks of training followed by one week of testing and de-loading. These blocks were categorized into several phases, including General Preparation (GP), Specific Preparation (SP), Competition Preparation (CP), and competition (Comp).

Assessment

The clinical examination was done for both groups at baseline and repeated every fourth day until the criteria for full participation was met and three outcome measures were recorded namely the Numeric Pain Rating Scale (NPRS) score during prone hamstring isometric contraction, length of hamstring tenderness (LHT), and active total knee extension (ATKE) range of motion (ROM) in degrees in supine lying with 90 degrees hip flexion.

The NPRS score of hamstring pain was recorded on the injured side during prone isometric contraction hold for five seconds at 90 degrees knee flexion. ATKE ROM was noted at an angle of perceived pain and restriction in the supine lying position with the hip at 90 degrees flexion wherein the athlete was instructed to actively extend the knee. The palpation method was utilized to identify the LHT area over the muscle or tendon to determine the location and extent of injury i.e. medial, lateral, proximal, or distal injury. These outcome measures were monitored on a regular basis to guide the criteria for return to full participation.

Interventions

During the initial stages of the injury, the athletes received relative rest with pain management strategies using various techniques including sports massage, ice, gentle stretching, and icing in both groups. Further management changed from the third day onwards as given below:

Group A from the third day onwards, technical training through sprints was introduced, with feedback provided by a coach. The sports physiotherapist present on the field led the identification of predisposing factors such as forward lean [5], spine or sacroiliac joint pathologies [14-16], flexibility in the hamstring and lumbopelvic complex [17-20], and poor conditioning, overtraining, undertraining, excessive stretching, and muscle imbalances [19,21-24]. Strength training was included based on training blocks/phases to maintain conditioning. Technical sprints were included along with strength training to prevent overstriding, with the help of mini hurdle runs over 50-60 meters. The cue was to "hit the foot under the hip, within the tolerable pain threshold." Soft tissue therapy and sports massage were used to reduce stiffness and pain post-training. Athletes were educated to train through pain within the tolerable pain threshold, and supportive care such as taping and strapping was provided if required. Once the pain was reduced, the technical aspect was transferred to flat running with 60 meters, including 20 meters of mini hurdles. The mini hurdles were gradually weaned off as the athlete became more aware of the technical aspects of sprints. Repeated sprints were given in the form of speed endurance, with 60 meters x 5 repetitions into three sets, with one-minute rest between reps and six minutes rest between sets. The decision of intensity increment was based on Table 2, in which the athletes were asked to do technical sprints as per the yellow zone derived within the center.

**Table 2:** Zones of training In zones of the training, athletes were educated to use this table to push through the intensity as the symptoms improved.

Red Zone	Yellow Zone	Green Zone
Pain >5/10 during and after and next morning	Pain <5/10 during and after but not next morning	No pain during, after and next morning
Pain on muscle contraction or weak contraction	Pain on muscle contraction but strong contraction	Pain free contraction or strong contraction
Pain with stretch or movement does not respond to therapy	Pain with stretch or movement but responds to therapy	No pain in stretch or movement
>5/10 pain with basic functional activity	<5/10 pain with basic functional activity	No pain

Group B, from the third day onwards, low-intensity and high-volume, isolated rehabilitation exercises were introduced, which consisted of strength exercises for the hamstring, gluteal muscles, and core. The progression was based on symptoms, starting with isometric exercises, then moving to concentric exercises, and finally to eccentric exercises. Once the participants in Group B met the criteria for a return to full participation, sprinting was introduced.

Zone for training

Zones of training were defined in collaboration with Coaches and Sports Physiotherapist for sprints which primarily included pain levels. The Traffic Light analogy is described in Table 2.

Criteria for return to full participation

Due to the absence of established clinical criteria for determining when track and field athletes are ready to resume full participation, our criteria were based on clinical outcome measures, which included assessments of the extent of the injury, loss of ROM, and pain provocation. These measures were evaluated every fourth day by a physiotherapist to track progress. The criteria were deemed met when the LHT (in cm) was reduced by more than 90%, the ATKE ROM was achieved at 95% or greater compared to the unaffected side, and the NPRS score on the resisted isometric test was less than 1/10.

Statistical analysis

Descriptive statistical analysis was introduced using Microsoft Excel 365 software (paid version, Microsoft® Corp., Redmond, WA), and the mean, standard deviation, and confidence interval were used for analysis. Google Forms (Google, Inc., Mountain View, CA) was used to collect data on a daily basis to analyze the effect of rehabilitation planning and modification in intensity. All Google Forms data were collected in Google Sheet format and later extracted the sheets between 2021 and 2023 and filters were created to track the improvement in various outcome measures, which were then copied into different Excel Sheets and it was analyzed.

## Results

Hamstring injuries

In Group A, eight total injured athletes had LHT of 9 cm (± 2.72, 95% CI 2.27), NPRS while performing the isometric test was 5.62 (± 1.06, 95% CI 0.88), and ATKE of 161.8° (± 7.12, 95% CI 5.95) shown in Table 3. In Group B, nine total injured athletes presented with LHT of 6.7 cm (± 2.1, 95% CI 1.62), NPRS while performing the isometric test was 6 (± 1.42, 95% CI 1.08), and ATKE was 168.6° (± 8.2, 95% CI 6.3) shown in Table 4, with no significant difference between the groups (p>0.05).

**Table 3:** Initial assessment of hamstring injuries in Group A Initial assessment of hamstring injuries in Group A with LHT of 9 cm (± 2.72, 95% CI 2.27), NPRS while performing the isometric test was 5.62 (± 1.06, 95% CI 0.88), and ATKE of 161.8° (± 7.12, 95% CI 5.95). NPRS: Numeric Pain Rating Scale; LHT: length of hamstring tenderness; CI: confidence interval

Injuries	Age (in years)	Height (cm)	Weight (Kgs)	Gender	Side of Hamstring Injury	Dominance	Palpation Tenderness (cm)	NPRS (out of 10)	Active Total Knee Extension (ATKE) Range of Motion (ROM)		
									Injured	% difference	Non-injured
Injury 1	22	179	63.2	Male	Right	Right	13	7	150	14.29%	175
Injury 2	31	193	93	Male	Right	Right	10	5	163	4.12%	170
Injury 3	31	193	93	Male	Right	Right	8	5	160	8.57%	175
Injury 4	18	187	63.7	Male	Right	Right	12	7	155	11.43%	175
Injury 5	19	163	50.4	Female	Right	Right	7	6	160	11.11%	180
Injury 6	19	163	49.9	Female	Right	Right	5	4	167	7.22%	180
Injury 7	21	174	50.4	Female	Left	Right	10	6	170	5.56%	180
Injury 8	31	193	93	Male	Left	Right	7	5	170	2.86%	175

**Table 4:** Initial assessment of hamstring injury in Group B Initial assessment of hamstring injuries in Group B demonstrated LHT of 6.7 cm (± 2.1, 95% CI 1.62), NPRS while performing the isometric test was 6 (± 1.42, 95% CI 1.08), and ATKE was 168.6° (± 8.2, 95% CI 6.3). NPRS: Numeric Pain Rating Scale; LHT: length of hamstring tenderness; CI: confidence interval

Injuries	Age (in years)	Height (cm)	Weight (Kgs)	Gender	Side of Injury	Dominance	Palpation Tenderness	NPRS (out of 10)	Active Total Knee Extension (ATKE) Range of Motion (ROM)		
									injured	% diff of normal	Non-injured
Injury 1	20	179	67.7	Male	left	Right	7	7	165	2.94%	170
Injury 2	19	162	55.3	Male	left	Right	10	9	150	14.29%	175
Injury 3	19	174	56.8	Female	Left	Right	5	6	175	2.78%	180
Injury 4	20	171	57.2	Male	Right	Right	8	5	168	4.00%	175
Injury 5	19	174	56.8	Female	left	Right	5	5	175	2.78%	180
Injury 6	18	174	64	Male	Right	Right	10	7	170	2.86%	175
Injury 7	30	166	65.8	Female	Right	Right	5	5	175	2.78%	180
Injury 8	19	174	56.8	Female	Right	Right	5	5	175	2.78%	180
Injury 9	21	154	53.1	Female	Right	Right	6	5	165	2.94%	170

Injuries reported in different blocks

The season was structured in different training blocks/phases as mentioned earlier, and different blocks/phases reported hamstring injuries which is described in Table 5.

**Table 5:** Injuries reported in different training blocks/phases Different blocks/phases of training in which hamstring injuries were reported. This includes GP, SP, and CP.

Blocks/Phases	Hamstring Injuries
General Preparation (GP)	6
Specific Preparation (SP)	4
Competition Preparation (CP)	7
Total	17

Both the groups exhibited considerable improvements in LHT, NPRS with the resisted isometric test, and ATKE with a p value of less than 0.05 as shown in Table 6. Group A showed significant improvement between the 24th and 28th day post-injury. LHT improved from 9 ± 2.7 (95% CI 2.27) to 0.15 ± 0.3 (95% CI 0.62), NPRS with resisted isometric test improved from 5.6 ± 1.09 (95% CI 0.88) to 0.6 ± 0.5 (95% CI 0.63), and ATKE improved from 161.8 ± 7.1 (95% CI 5.95) to 175.4 ± 2 (95% CI 2.3). Group B showed significant improvements between the 32nd and 36th day post-injury. LHT improved from 6.8 ± 2.1 (95% CI 1.62) to 0.6 ± 0.7 (95% CI 0.55), NPRS with resisted isometric test improving from 6 ± 1.4 (95% CI 1.08) to 0.8 ± 0.7 (95% CI 0.51), and ATKE improving from 168.7 ± 8.2 (95% CI 6.3) to 178.7 ± 2.7 (95% CI 2.06). These results suggest that Group A returned to full participation earlier than Group B. The baseline data demonstrates that Group A experienced significantly greater LTH and ATKE (p <0.05). However, the NPRS scores reported during the resisted isometric test were similar between the groups (p >0.05).

**Table 6:** Results LHT: length of hamstring tenderness; NPRS: Numeric Pain Rating Scale, ATKE (ROM): active total knee extension range of motion; # indicates mean; * indicates standard deviation (+ or -), CI: confidence interval at 95%; p value of less than 0.05 is taken as statistically significant. In ATKE ROM, the baseline data for Groups A and B showed 8.1% and 4.2%, and the final result showed a 1.2% and 1.8% difference compared to the uninjured side respectively.

Objective Measures	Group A					Group B				
	Baseline		Final		P value	Baseline		Final		P value
LHT (in cm)	9^#^ ± 2.7*	95% CI 2.27	0.125^#^ ± 0.3*	95% CI 0.62	P <0.05	6.8^#^ ± 2.1*	95% CI 1.62	0.6^#^ ± 0.7*	95% CI 0.55	P <0.05
NPRS (out of 10)	5.6^#^ ± 1.09*	95% CI 0.88	0.6^#^ ± 0.5*	95% CI 0.63	P <0.05	6^#^ ± 1.4*	95% CI 1.08	0.8^#^ ± 0.7*	95% CI 0.51	P <0.05
ATKE (ROM)	161.8^#^ ± 7.1* (8.1% diff of uninjured side)	95% CI 5.95	175.4^#^ ± 2* (1.2% diff of uninjured side)	95% CI 2.3	P < 0.05	168.7^#^ ± 8.2* (4.2% diff of uninjured side)	95% CI 6.3	178.7^#^ ± 2.7* (1.8% diff of uninjured side)	95% CI 2.06	P < 0.05

The difference in the time taken for full participation to be resumed between Group A and Group B was notable, with Group A requiring an average of 3.55 weeks (with a standard deviation of 1.22 and a 95% confidence interval of 1.08) compared to Group B's 4.53 weeks (with a standard deviation of 1.98 and a 95% confidence interval of 1.52). Group A was able to resume full participation one week prior to Group B.

**Figure 1:**
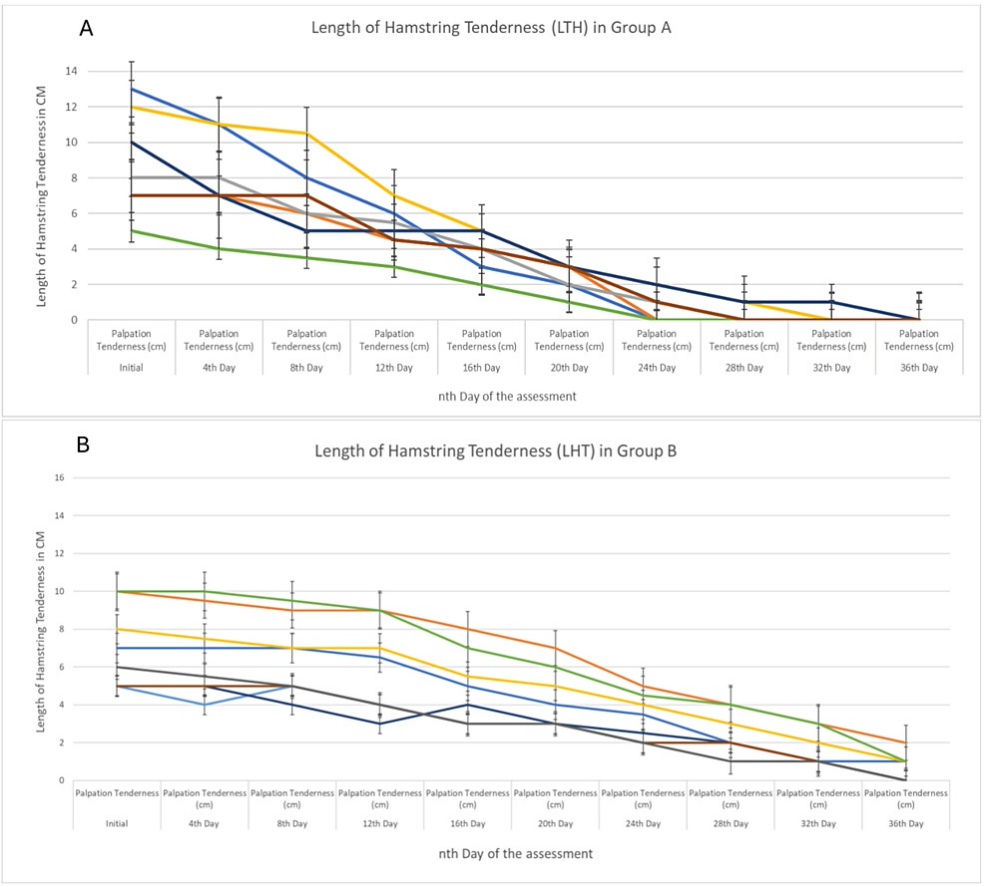
Length of hamstring tenderness in Groups A and B In Figure 1A, the graph represents the length of hamstring tenderness, which improved between the 24th and 28th day from the initial assessment. However, in Figure 1B, the improvement was recorded between the 32nd and 36th day from the initial assessment. Error bars represent the 95% confidence interval of the data.

Figure 1 shows the LHT in Groups A and B, and reduction over a period of every fourth day until it achieved the desired return to full participation criteria.

**Figure 2:**
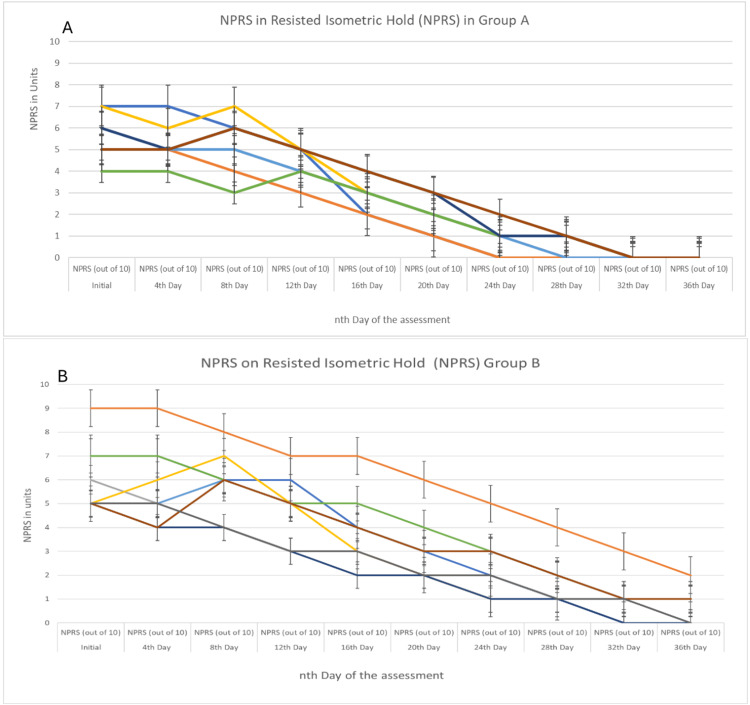
NPRS in resisted isometric hold in Groups A and B Figure 2A represents the NPRS score in Group A and recorded the improvement between the 24th and 28th day; however, Figure 2B recorded the improvement between the 32nd and 36th day which represents the NPRS score in Group B. The error bars represent a 95% confidence interval. NPRS: Numeric Pain Rating Scale

Figure 2 shows NPRS in resisted isometric hold in Groups A and B, and reduction over a period of every fourth day until it achieved the desired return to full participation criteria.

**Figure 3:**
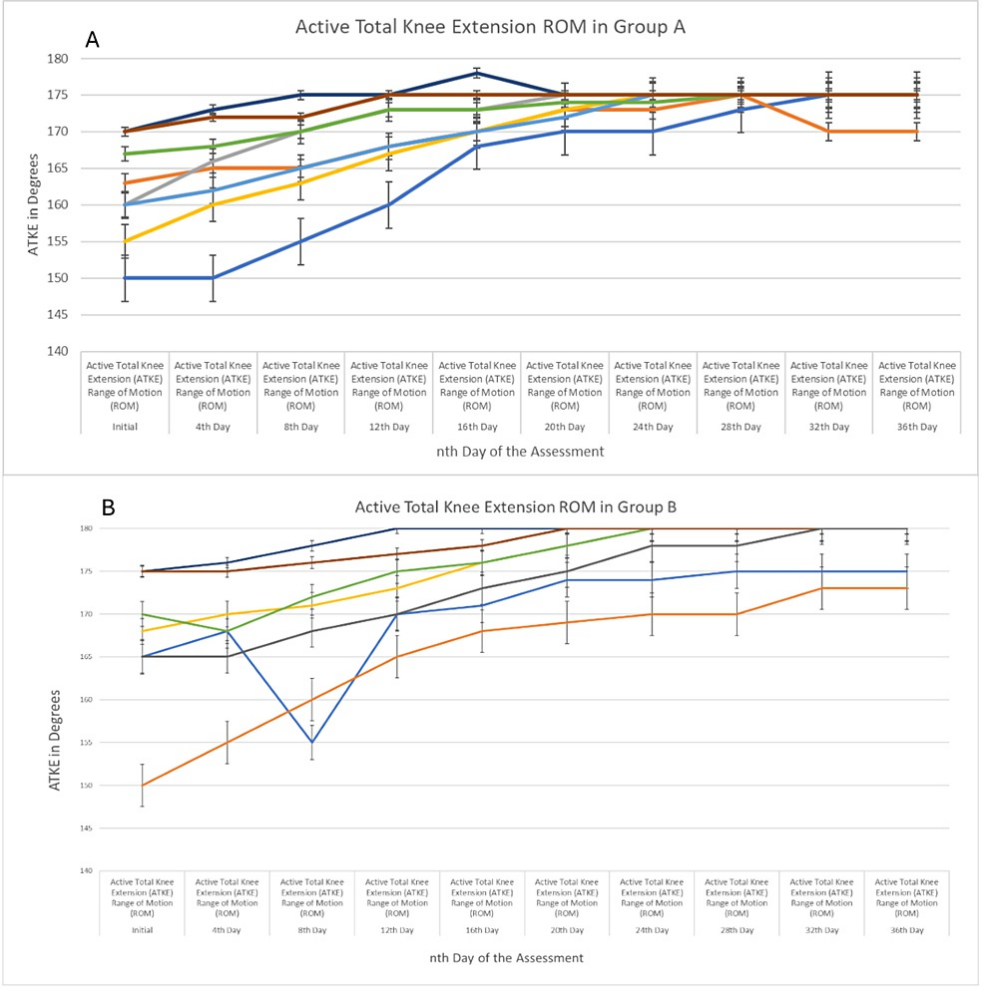
Active total knee extension (ATKE) range of motion (ROM) in Groups A and B Active total knee extension (ATKE) range of motion (ROM) was measured using a goniometer and the maximum ROM taken was 180 degrees. Figure 3A shows improvement in the overall ROM in Group A which was recorded between the 24th and 28th day; however, in Figure 3B, the improvement was recorded between the 32nd and 36th day post-initial assessment. Error bars represent the 95% confidence interval of the data.

Figure 3 shows ATKE ROM in Groups A and B, and improvement over a period of every fourth day until it achieved the desired return to full participation criteria.

Corresponding to injury reported during the different blocks and reinjury or hamstring tightness reported post-competition are shown in Table 7. In Group B we recorded two re-injury which corresponded to SP and CP, however, the tightness reported post-competition was three and six in Groups A and B respectively as shown in Table 7.

**Table 7:** Hamstring reinjury and tightness during competition Hamstring injury and tightness were reported during the competition from both Groups A and B.

Blocks/Phases	Hamstring Injuries	Group A	Group B	Hamstring Reinjury		Hamstring Tightness	
				A	B	A	B
General Preparation (GP)	6	3	3	-	-	2	1
Specific Preparation (SP)	4	2	2	-	1	1	1
Competition Preparation (CP)	7	3	4	-	1	-	4
Total	17	8	9	0	2	3	6

## Discussion

Hamstring injuries are a very common non-contact type of muscle injury, which negatively affects performance if not managed appropriately, with the major factor being a risk of reinjury and loss of training time. This article demonstrated incorporating technical sprints from the early stages of hamstring injury rehabilitation and its effect on the time taken for return to full participation, performance, signs and symptoms, and chances of re-injury. In track and field events, the sports-specific rehabilitation protocol for hamstring injuries is imperative due to the demanding nature of the sport. This study compared outcomes among two groups to evaluate the influence of two types of hamstring rehabilitation protocol. Both the groups demonstrated significant improvement in all three outcome measures - LHT (in cm), ATKE ROM, and NPRS score on resisted isometric test. However, Group A returned to full participation between the 24th and 28th days whereas in Group B it took about the 32nd to 36th days. In Group A, the technical sprints were included from the first week of hamstring injury rehabilitation which could have promoted the local tissue health [26], hence developing confidence among athletes to perform at their best effort. Evidence has suggested that EMG activity in the hamstring is highest during sprinting and has been incorporated for hamstring conditioning and for the prevention of hamstring injuries as compared to isolated hamstring exercises [27]. Hickey et al. have demonstrated in their article that working through pain aids in increasing the fascicle length of the hamstring muscle [28] and results in improved eccentric muscle capacity which is imperative in sprinting and a most vulnerable risk factor for a hamstring injury. This physiological change in tissue might be a probable reason that helped Group A participants for early return to full participation. Additionally, it was also observed that the reinjury among Group B athletes corresponds to the specific preparation and competition preparation phase of training. This may indicate that athletes had less time to prepare for the competition after an injury; however, athletes who complained of hamstring tightness may be suggestive of poor hamstring conditioning or capacity to withstand high-intensity sprinting. In Group A, the early inclusion of technical sprints helped in hamstring conditioning, which might have prevented reinjury and symptoms during competition.

This study is limited by the small sample size and the grade of the hamstring injury which was not determined based on MRI [29], rather only clinical examination findings were utilized to identify the extent of injury.

Based on the results, this study concludes that early inclusion of technical sprints facilitates early return to full participation and hence recommends it to be incorporated as part of hamstring injury rehabilitation, however, this is a preliminary study, more detailed study is required with MRI based grading [29] system to understand the implications of intervention. Currently, in practice, there are no established prevention methods for hamstring injuries caused by high-intensity sprinting in track and field sports. However, as sports science continues to advance, it is important for coaches, strength and conditioning trainers, and sports physiotherapists to work together to improve sprinting mechanics to reduce time loss and help athletes return to full participation as soon as possible.

## Conclusions

To the best of our understanding, this is the initial investigation to explore the utilization of technical sprints for hamstring rehabilitation in hamstring injury. The findings indicate that early technical sprints, centered on the mechanical aspects of sprinting, can facilitate an early return to full participation without apprehension and without significant reinjury rates compared to low-intensity high-volume rehabilitation exercises.
